# miR-181a Post-Transcriptionally Downregulates Oncogenic RalA and Contributes to Growth Inhibition and Apoptosis in Chronic Myelogenous Leukemia (CML)

**DOI:** 10.1371/journal.pone.0032834

**Published:** 2012-03-19

**Authors:** Jia Fei, Yumin Li, Xuejiao Zhu, Xiaochuang Luo

**Affiliations:** Department of Biochemistry and Molecular Biology, Medical College of Jinan University, Guangzhou, China; Roswell Park Cancer Institute, United States of America

## Abstract

MicroRNAs (miRNAs) are a class of short RNAs that regulate gene expression through either translational repression or mRNA cleavage. miRNA-181a (miR-181a), one of the many miRNAs conserved among vertebrates, is differentially expressed in a variety of leukemia. However, its function in leukemia, particularly chronic myelogenous leukemia (CML), is poorly understood. Here we have reported the identification of miR-181a targets by combining TargetScan software prediction and expression profiling through overexpression of miR-181a mimic in leukemic K562 cells. Four overlapping genes were found to be the likely targets of miR-181a. Among the four genes, RalA is a downstream molecule of bcr-abl fusion protein in ras signaling pathway. However, its role in CML remains elusive. Luciferase reporter and Western blot assays confirmed that RalA is a direct target of miR-181a. overexpression of miR-181a effectively suppresses cell growth and induces G2-phase arrest and apoptosis partially by targeting RalA in leukemic K562 cells. Using the KEGG database combined with recent publications, downstream signaling pathway of RalA was graphed by cytoscape software. Therefore, our study is the first to report that RalA is directly regulated by miR-181a and plays an important role in CML. The approach of computational prediction combined with expression profiling might be valuable for the identification of miRNA targets in animal.

## Introduction

MicroRNAs (miRNAs) are a new class of endogenous noncoding small RNA molecules that have been shown to be important regulators of gene expression in cells. miRNAs post-transcriptionally regulate gene expression by either cleavage or repression of mRNA through binding to the 3′-untranslated region (3′-UTR) of the target genes [1],[2]. Bioinformatics prediction indicates that 30% of all the genes are regulated by miRNAs [3]. Currently, more than 1000 miRNAs have been identified in humans [4]. They are known to be involved in a variety of functions in development, cell proliferation, apoptosis, differentiation, and tumorigenesis [1],[2]. Thus, miRNA functional identification has become one of the most attractive research fields in biomedicine. Unfortunately, most of these miRNAs have unknown functions [5]. One major obstacle is to identify the targets regulated by miRNAs. Since there is partial complementarily between miRNAs and their targets in animal cells, the identification of specific target genes for a given miRNA is still a big challenge in our understanding of the miRNA functions. Computation-based approaches for miRNA gene identification and miRNA target prediction are considered as indispensable in miRNA research. Several computational approaches have been developed for the prediction of miRNA targets.

Computational algorithms based on the factors, such as free binding energy or sequence conservation, have been used to predict miRNA targets. The prediction of miRNA targets has been based on putative binding sites within the 3′-UTRs of the transcripts [6]. The efficacy of the computational approaches to locate and rank the potential binding sites is supported by the relatively high degree of miRNA complementarity to the experimentally determined binding sites. Based on such predictions, up to one-third of all mammalian mRNAs appear to be under miRNA regulation.

Despite the subsequent identification of hundreds of miRNAs in a variety of species, only a handful of targets have been confirmed experimentally [7]. Owing to the imperfect complementarity between any given miRNA and its targets, computational predictions are often hindered by false-positives. Therefore, effective experimental techniques to validate the predictions are crucial for the testing and fine-tuning of computational algorithms [8],[9].

It was previously presumed that most miRNAs regulate the target gene expression via mRNA degradation in plants [10], whereas in animals, miRNAs are considered to suppress the target gene translation [2]. However, recent studies have convincingly demonstrated that the levels of animal mRNAs are also altered if the target-binding sites have perfect sequence complementarity to the miRNA [11]. Furthermore, miR-16 has been shown to mediate mRNA degradation, even if the binding site has only a partial sequence match [12]. Currently, mounting evidence has shown that animal miRNAs can reduce the mRNA levels via mRNA degradation, and that extensive complementarity is not always required [13],[14]. These findings indicate that it is more convenient for miRNA target validation to monitor mRNA changes using a high-throughput experimental approach when compared with changes in protein expression.

Overexpression of miRNA in cells has been reported to lead to the downregulation of a large number of transcripts, as revealed by the microarrays [9],[15],[16]. A significant enrichment of miRNA complementary sequences in these downregulated genes has been observed, implying that it might be possible to use microarrays to simultaneously identify a large number of miRNA targets.

miRNA-181a (miR-181), one of the many miRNAs conserved among the vertebrates, is preferentially expressed in the B lymphocytes of bone marrow, and its ectopic expression in hematopoietic stem/progenitor cells modulates the blood cell development [17]. Recent studies have shown that miR-181a is differentially expressed in a variety of leukemia [18],[19],[20]. However, little is known about the role of miR-181a in chronic myeloid leukemia (CML).

In our previous research, we found that the expression of miR-181a is very low such that it cannot be detected by quantitative real-time PCR in K562 cells, indicating that downregulation of miR-180a might play an important role in leukemogenesis. To elucidate the function of miR-181a in CML, it is important to identify its target genes. In the present study, we have combined computational prediction and expression profiling methods to systematically identify the miR-181a targets following overexpression of miR-181a mimic. Our data show that RalA is a direct target gene of miR-181a and associated with cell proliferation, G2-phase arrest and apoptosis in CML.

## Results

### miR-181a target prediction

TargetScan, the first method employed for human miRNA target prediction using mouse, rat, and fish genomes for conservation analysis [7], was used to predict miR-181a targets. A total of 691 genes were predicted to be putative targets of miR-181a. These genes, including tumor suppressor genes and oncogenes, are involved in various aspects of functions implicated in cancer, including control of cell proliferation, apoptosis, signal transduction, transcription regulation, immunity and defense (Table S1).

### miR-181a transfection microarray

miR-181a duplex molecule RNA (mimic) was transfected into the K562 cells. Gene expression profiling was conducted 48 h post-transfection. From the 20173 screened genes, 248 genes were differentially expressed between control and miR-181a transfected cells. Among them, 65 genes showed increased expression, whereas 183 genes showed decreased expression compared to the control. As miRNAs are known to cause degradation or translational repression of target mRNAs, we focused on the genes that are downregulated in miR-181a-transfected cells. The majority of the downregulated genes were found to be involved in the regulation of cell cycle and proliferation, DNA-binding and transcription, metabolism, signal transduction, development, etc. (Table S2.), suggesting that miR-181a may be involved in various aspects of leukemia.

### Characterization of the downregulated miR-181a targets

**Figure 1:**
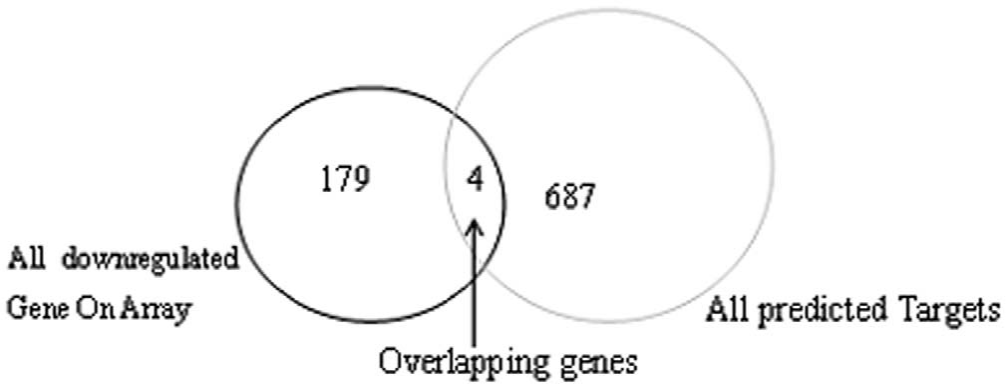
Downregulated genes upon miR-181a overexpression. miR-181a targets were identified by combining TargetScan software prediction and expression profiling. The four genes identified by both methods were considered as candidate targets of miR-181a in K562 cells.

Four of the predicted targets were subsequently confirmed to be downregulated by miR-181a in K562 cells. One of them, RalA, is a v-ral simian leukemia viral oncogene homolog A (ras-related) (Table 1) involved in tumorigenesis and cancer invasion [22],[23]. Thus we next wanted to experimentally validate whether RalA is a direct target of miR-181a (Figure 1).

**Table 1:** Characterization of RalA gene.

Entrez gene ID	Gene Name	Ratio (Cy5/Cy3)	*p*-value LogRatio	Total contextscore	GeneDescription
5898	RalA	0.6862	0.0013	−0.26	v-ral simian leukemia viraloncogene homolog A (ras-related)

*p*-value LogRatio was used as the criteria to estimate differences in the gene expression on array. The total context score was used to assess whether there was significance in the predicted miR-181a targets in the downregulated genes.

### The RalA-3′-UTR is a target for miR-181a

**Figure 2:**
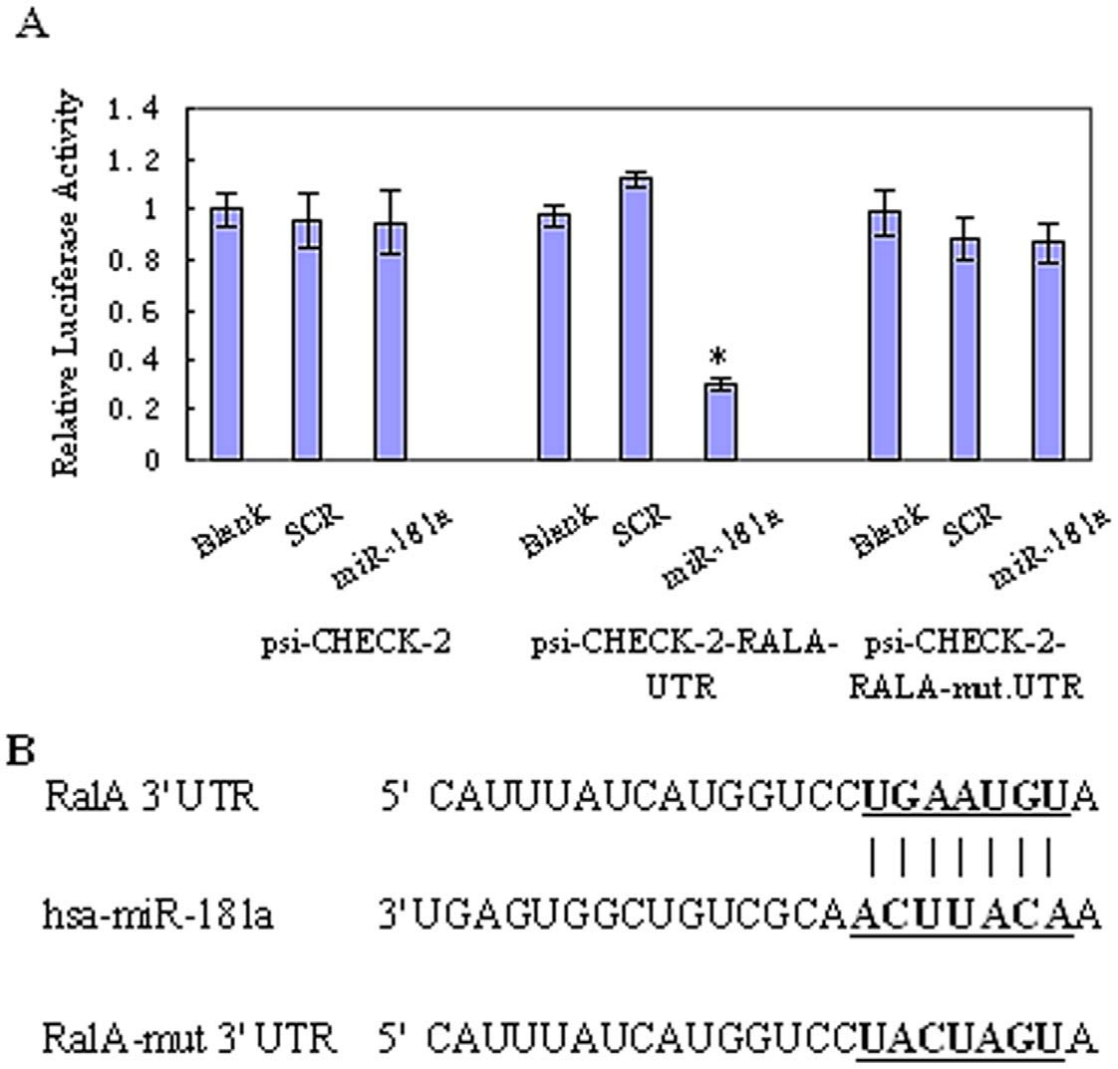
miR-181a-directed repression of Renilla luciferase reporter genes containing RalA-3′-UTR segments. K562 cells were cotransfected with scrambled RNA or miR-181a together with RalA-3′-UTR or RalA-mut-3′-UTR luciferase reporter in the presence of firefly luciferase reporter plasmid as indicated. Renilla luciferase activity and firefly luciferase activity were measured by dual-luciferase reporter assay (Promega). Renilla luciferase activity was normalized to firefly luciferase activity. The data represent the mean value of three independent experiments. (A) overexpression of miR-181a significantly repressed Renilla luciferase activity in cells transfected with RalA-3′-UTR, but not RalA-mut-3′-UTR vector; *p<0.01. (B) Human RalA-3′-UTR and its miR-181a target site predicted by TargetScan software. Mut: contains 7-base-mutation at the miR-181a target region.

We first determined whether the 3′-UTR of the RalA gene contains functional miR-181a targeting sequence(s), and whether direct interaction between the two takes place. To this end, we constructed dual-luciferase reporter a pair of plasmids containing two tandem miR-181a recognition sequences from the 3′-UTR of RalA mRNA and the mutated recognition sequences immediately downstream of the luciferase gene (psi-CHECK-2-RalA-UTR and psi-CHECK-2-RalA-mut-UTR reporter, respectively). Using these plasmids, we performed dual-luciferase reporter assays. As shown in Figure 2AB, transfection with miR-181a significantly decreased the activity of psi-CHECK-2-RalA-UTR, but not psi-CHECK-2-RalA-mut-UTR, reporter in the K562 cells. Taken together, these data suggest that the RalA might be a direct target of miR-181a in K562 cells.

### miR-181a downregulates RalA mRNA and protein level

**Figure 3:**
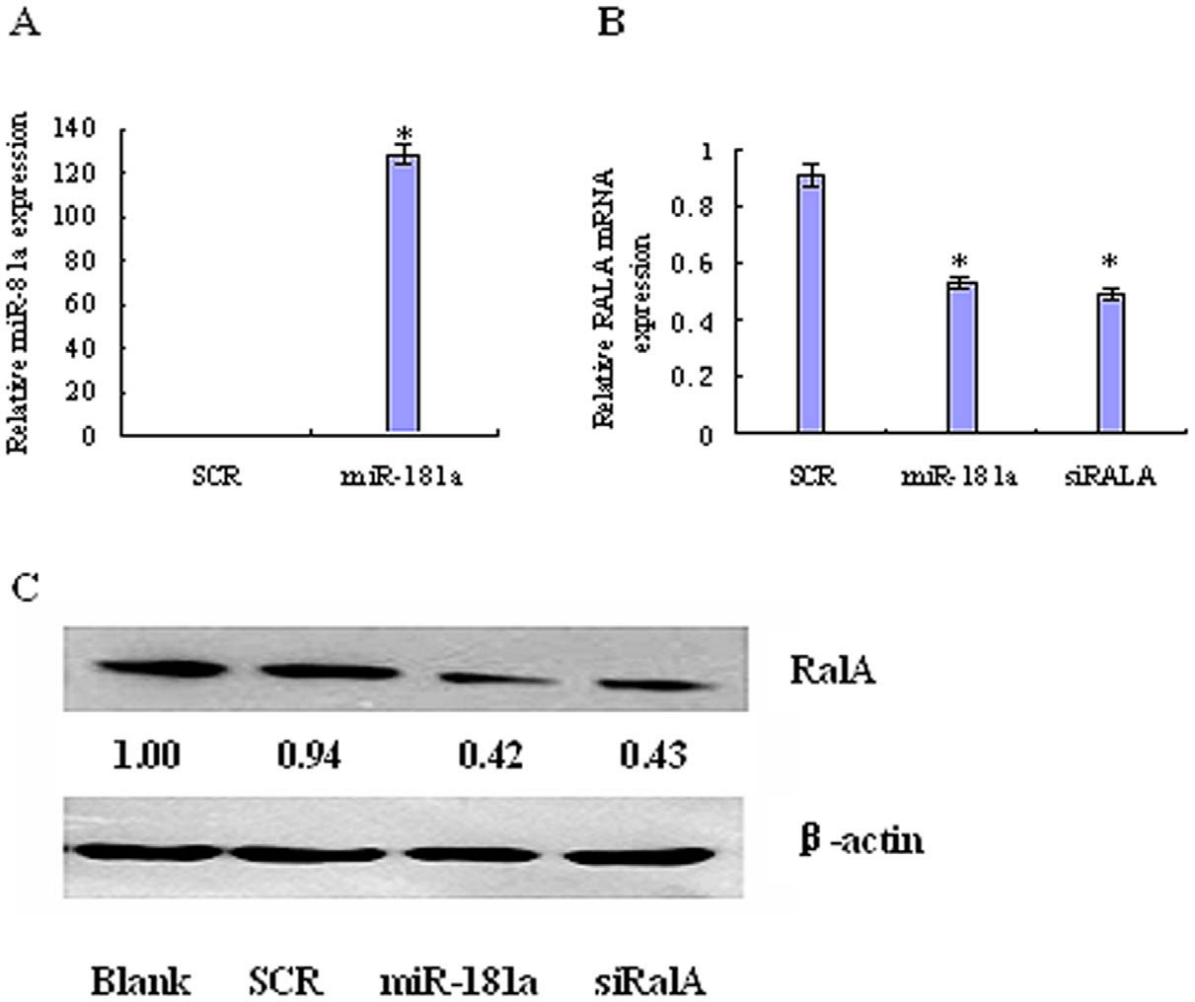
miR-181a regulates RalA expression at the post-transcriptional level. K562 cells were transfected with scrambled RNA, miR-181a, or RalA siRNA and harvested 48 h after transfection. Total RNA was isolated and analyzed for the expression of miR-181a (A), RalA mRNA (B) and RalA protein (C) using qPCR and Western blot, respectively. *p<0.01, when compared with controls.

Next, we tested whether miR-180a can downregulates RalA mRNA in K562 cells. The miRNA transfection in K562 cells was efficient as the level of miR-181a was increased by 140-fold in miR-181a transfected cells compared to scrambled RNA transfected cells (Figure 3A). Subsequently, we evaluated the effects of miR-181a on the expression of RalA protein and mRNA using immunoblot and RT-real-time PCR analysis. As shown in Fig. 3B, overexpression of miR-181a resulted in a moderate reduction in the RalA mRNA levels and reduced RalA protein level. This reduction if comparable to that mediated by transfection of RalA siRNA in K562 cells (Figure 3C).

### miR-181a suppresses cell growth partially by targeting RalA

**Figure 4:**
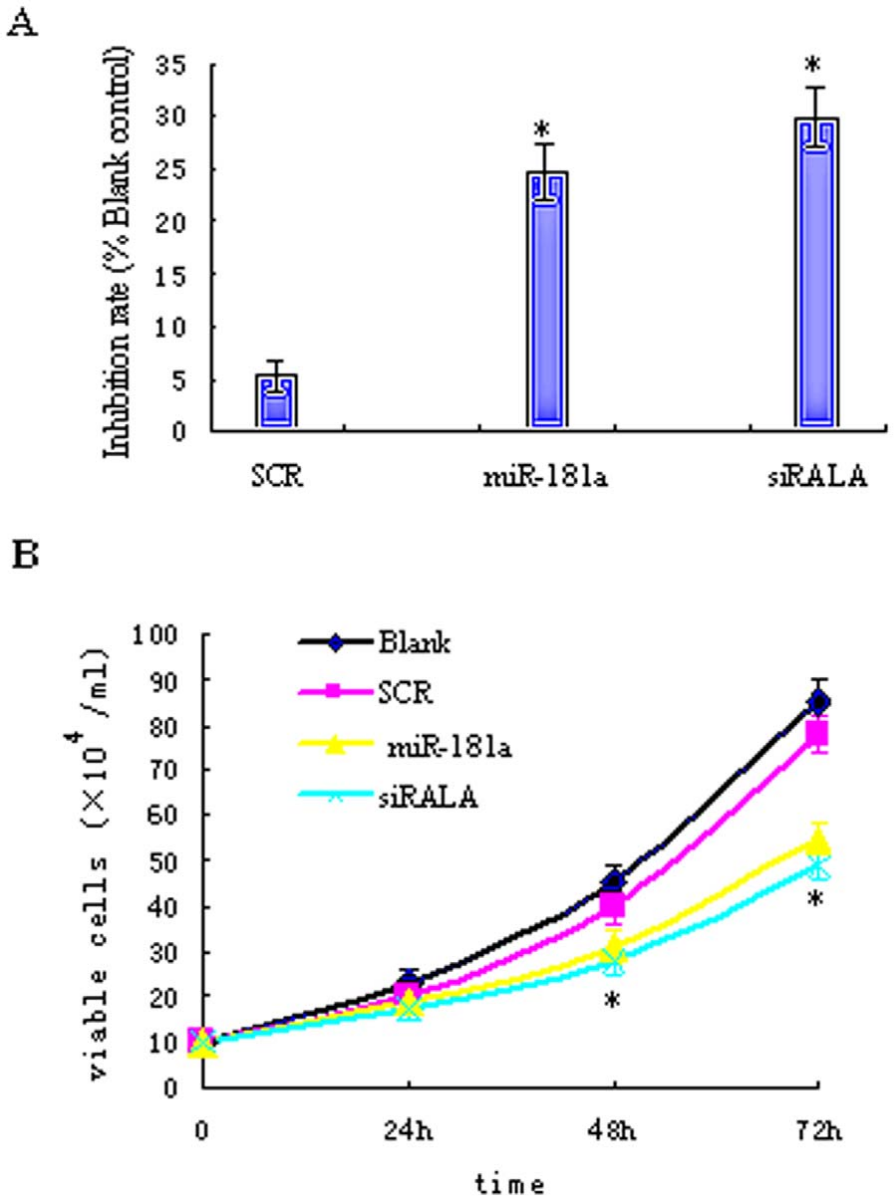
miR-181a inhibits K562 cells growth partially by targeting RalA. K562 cells were transfected with scrambled RNA, miR-181a, or RalA siRNA. Viability of the cells was assessed by MTT (**A**) and trypan blue exclusion assay (**B**) in triplicates. Both miR-181a and siRNA RalA effectively inhibited cell viability in a time-dependent manner. *p<0.01, when compared with blank or Scramble controls.

To determine whether miR-181a affects cell growth, we transfected K562 cells with scrambled RNA, miR-181a or RalA siRNA followed by assay. As shown in Fig. 4, both miR-181a and RalA siRNA effectively inhibited cell viability in a time-dependent manner. RalA siRNA seemed to have stronger effects, although not statistically different, than miR-181a (no difference in statistics was observed). These findings strongly suggest that miR-181a suppressed cell growth at least partially by targeting RalA (*p<0.01) (Figure 4AB).

### miR-181a induces G2-phase arrest partially by targeting RalA

**Figure 5:**
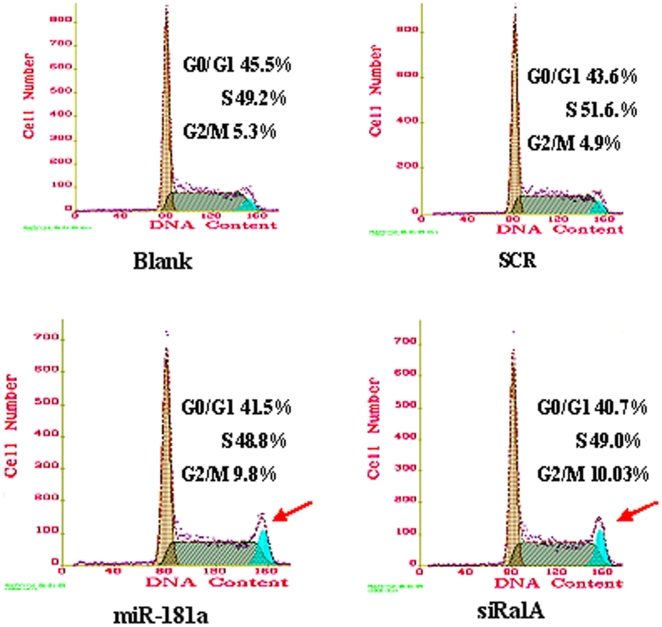
miR-181a regulates cell cycles partially by targeting RalA. K562 cells were transfected with scrambled RNA, miR-181a, or RalA siRNA and harvested 48 h post-transfection. The cells were stained with PI solution and analyzed using flow cytometry. Both miR-181a and RalA siRNA significantly induced G2-phase arrest. *p<0.01, when compared with blank or SCR controls.

To elucidate the influence of miR-181a on cell cycles, K562 cells transfected with above scramble, miR-181a or RalA siRNA were subjected to cell cycle analysis. As shown in Figure 5, overexpression of miR-181a resulted in an accumulation of cells in G2/M. Interestingly, siRNA RalA produced almost identical effects on cell cycles (Figure 5). These results suggest that miR-181a-induced G2/M arrest is at least partially due to its targeting of the RalA gene.

### miR-181a promotes apoptosis partially by targeting RalA

**Figure 6:**
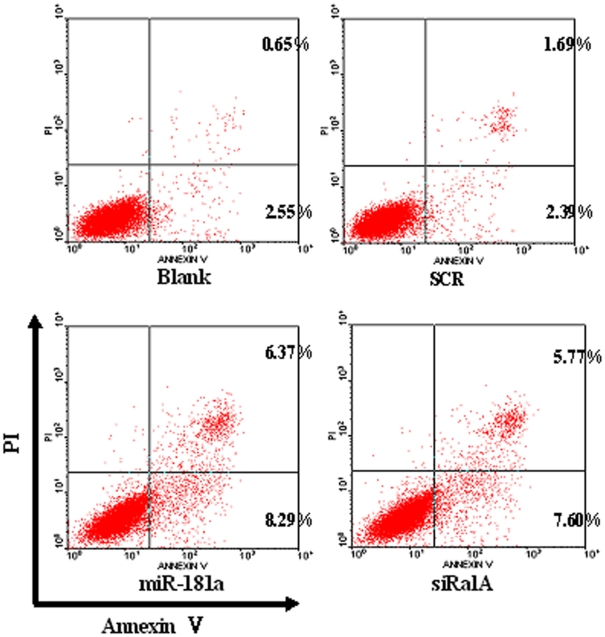
miR-181a promotes apoptosis partially by targeting RalA. K562 cells were transfected with scrambled RNA, miR-181a, or RalA siRNA and harvested 48 h after transfection. The cells were stained with FITC-conjugated annexin V and PI, followed by flow cytometry analysis. Annexin V-positive and PI negative cells represent apoptotic cells. *p<0.01, when compared with blank or SCR controls.

Finally, we determined whether miR-181a is involved in apoptosis. K562 cells transfected with scrambled, miR-181a or RalA siRNA were stained with Annexin V and PI followed by flow cytometry. As shown in Fig. 6, overexpression of miR-181a significantly decreased apoptosis in these cells. The results demonstrated that miR-181a induced cell apoptosis at least partially by targeting RalA.

### Pathway of RalA

**Figure 7:**
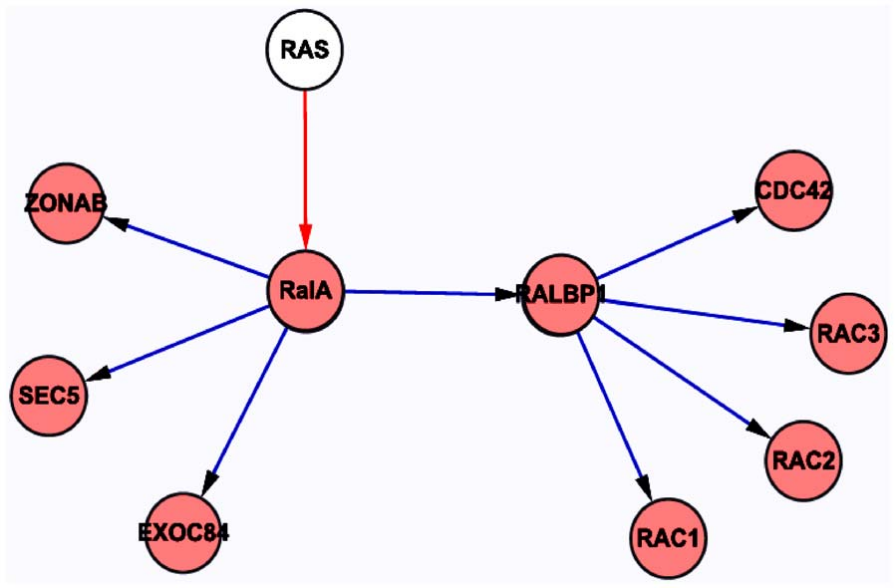
KEGG pathway analysis of RalA downstream regulated genes. The list of known potential targets of RalA graphed using cytoscape.

Using the KEGG database, combined with recent publication, we analyzed genes in downstream of RalA signaling pathways, including the Rac-family GTPase-activating protein RLIP (also known as RLIP76 and ralA binding protein 1 (RALBP1)), CDC42(cell division cycle 42), RAC1 (ras-related C3 botulinum toxin substrate 1), RAC2, RAC3, the Y-box transcription factor ZO-1-associated nucleic acidbinding protein (ZONAB, also known as cold shock domain protein A (CSDA)), and two subunits of the exocyst complex, SEC5 (also known as exocyst complex component 2 (EXOC2)) and EXO84 (also known as EXOC8), which are involved in regulating cell proliferation, apoptosis and migration. The result was graphed using cytoscape to show the interactions between RalA and its potential targets(Figure 7).

## Discussion

Prediction of animal miRNA targets is very challenging owing to the imperfect pairing of a given miRNA with its targets as well as the fact that the gene expression is in a tissue-specific and/or time-specific manner [1]. This predictive may have a high false-positive rate that may require experimental verification.

While mRNA microarray analysis following miRNA inhibition or overexpression is a relatively simple and robust method for target identification, this approach, as per definition, cannot identify mRNAs subjected exclusively to the translational repression. In other words, this method may produce a high false-negative result. However, this disadvantage may not be as severe as initially thought, as the recent data suggest that it is a common mechanism for animal miRNAs to downregulate target mRNA expression and that the majority of miRNA regulation can be detected at the mRNA levels [16]. This allows to identify and experimental validation of target genes using mRNA expression profiling. Hence, we attempted to identify miRNA-181a target genes using a combination of computational prediction and expression profiling. As a result, we identified four candidate targets of miR-181a.

Among them, RalA is found to play important roles in tumorigenesis and cancer invasion [22],[23]. Our dual-luciferase reporter and Western blot assays confirmed that RalA contains a miR-181 binding site at its 3′-UTR and is directly regulated by miR-181a (Figure 2A, 3C). overexpression of miR-181a significantly downregulated the RalA mRNA level (Figure 3B), Suggesting that RalA is an authentic miR-181a target.

RalA and its isoform RalB are multifunctional GTPases involved in the control of cell proliferation, oncogenic transformation, and membrane trafficking [22],[24]. Although RalA and RalB share about 85% of their amino acid sequences, the functions of the two isoforms are only partially overlapping. RalA and RalB have antagonistic effects on cancer cell migration, but possess overlapping functions in cell growth [25]. Lim et al. reported that RalA and RalB are more commonly activated in the pancreatic tumor tissue and that the RalA function is critical to tumor initiation, whereas RalB function is more important for tumor metastasis in the tested cell lines [26].

RalA, one of the Ral family small G proteins, has been implicated in tumorigenesis, invasion, and metastasis of a variety of solid tumors [22]. Thus, activation of RalA signaling appears to be a critical step in the Ras-induced transformation and tumorigenesis [23]. Wang et al. showed that RalA contributes to liver malignant transformation and could be a potential tumor marker in hepatocellular carcinoma (HCC) [27]. Shi et al. reported that miR-181a and miR-181b function as tumor suppressors by inducing cell growth inhibition apoptosis, and repression of invasion in glioma cells [28].

CML results from the transformation of primitive hematopoietic cells by the Bcr/Abl gene product. BCR-ABL regulates activation of many mitogenic and pro-survival pathways, including the PI 3′K/AKT/mTOR pathway that controls various effectors. Recent research showed that AMPK and/or AXL, overexpression in Imatinib (IM)-resistant CML cell lines and patients, might be novel target in treatment of refractory CML [29],[30]. In our KEGG analysis, several important genes were shown downstream to the RalA signaling pathways, including RALBP1, CDC42, RAC1, RAC2, RAC3, ZONAB, and SEC5 and EXO84 (Figure 7), further supporting the notion that RalA function is critical to tumor initiation.

Further research demonstrates that Bcr-Abl oncoprotein binds directly to activators of the Ras signaling pathway and effector molecules of RalA, such as CDC42 and Grb2 in ras signaling pathway, play a major role in Bcr-Abl-induced leukemogenesis [31],[32],[33]. Therefore, RalA, as one small GTPase molecule of the Ras network, might also play an important role in oncogenesis of CML.

It has clearly been demonstrated that the most primitive stem cells are refractory to all TKIs used in clinical practice. Some signaling pathways specific to LSCs have been successfully identified in CML stem cells. Wnt/β-catenin, Sonic Hedgehog, Notch and Alox5 signaling pathways play crucial roles in the maintenance of stem cell functions. The inhibition of these signaling pathways specifically impairs LSC functions without affecting normal HSCs. The role of ras signaling pathway in CML stem cells is poor understood [34].

However, the role of miR-181a in CML has not been well characterized. In agreement with Ramkissoon et al. [35], our data revealed that miR-181a was hardly detected in K562 cells using Northern blot. Our further data showed that overexpression of miR-181a in K562 cells significantly suppressed cell growth (Figure 4) and induced G2-phase arrest (Figure 5). Interestingly, ablation of RalA expression by siRNA also led to almost identical effects as those produced by miR-181a. Therefore, our results suggest that miR-181a functions to suppress cell growth by, at least partially, targeting RalA.

miR-181 has also been implicated in other leukemias. For example, Pekarsky et al. showed that miR-181b could inhibit chronic lymphocytic leukemia (CLL) cells by targeting oncogenic Tcl1 [18]. Moreover, miR-181 was presumed to be one of the pathogenic factor of CLL [19]. Recently, another work demonstrated that miRNA-181 family members were associated with event-free survival in acute myeloid leukemia (AML) and that downregulation of the miRNA-181 family contributes to an aggressive leukemia phenotype through mechanisms associated with the activation of pathways controlled by toll-like receptors and interleukin-1β [20].

In conclusion, our work revealed that overexpression of miR-181a causes cell growth inhibition, G2-phase arrest and apoptosis through a mechanism that at least partially targets RalA in K562 cells, suggesting that miR-181a might function as a tumor suppressor in hematopoietic cells. Our study also indicates that a combination of computational prediction and expression profiling might be an applicable choice for the identification of miRNA targets.

## Materials and Methods

### Cell line

K562 (chronic myelogenous leukemia) cell lines (from the Institute of Shanghai cell biology, China) were grown in a RPMI-1640 medium containing 10% fetal calf serum (FCS) at 37°C, in 5% CO_2_, humidified atmosphere (Thermo FORMA 3110).

### miRNA and siRNA transfections

miR-181a duplex (sense: 5′-AACAUUCAACGCUGUCGGUGAGU-3′; antisense: 5′-UCACCGACAGCGUU GAAUGUUGU-3′) and small interfering RNA (siRNA) against RalA (siRalA) (sense: 5′-CGUGGAAACAUCUGCUAAATT-3′ and antisense: 5′-UUUAGCAGAU GUUUCCACGTA-3′) were synthesized by Shanghai GenePharma Company in China and stored at −20°C. The miR-181a RNA duplex (mimic) and scramble (Scr) control miRNA duplex (100 nM) were transfected into K562 cells using Oligofectamine™. Breifly, RNAs were diluted in optiMEM medium (Invitrogen). The diluted miRNA duplex was then combined with diluted transfection reagent, followed by incubation at room temperature for 10 min. The mixture was dispensed into an empty 24-well plate (Costar). K562 cells were then added into each well at a concentration of 5×10^5^ cells per well. Total RNAs were extracted at 48 h posttransfection for microarray experiments. Additional transfections were performed to generate RNA samples for RT-PCR validations.

### Computational prediction of miR-181a targets

To identify the miR-181a targets, TargetScan software (version 4.0 July 2007) (7), available at http://genes.mit.edu/targetscan, was employed. This prediction combines thermodynamics-based modeling of RNA:RNA duplex interactions with comparative sequence analysis to predict the miRNA targets conserved across multiple genomes (Figure 2B). TargetScan requires perfect complementarity to the seed region of the miRNA, and subsequently extends these regions to unravel the complementarity outside the region. This results in filtering of many false-positives from the beginning of the prediction process. Subsequently, the predicted binding sites are tested for their thermodynamic stability. The context score (Table 1) for a specific site is the sum of the contribution of four features: site-type contribution, 3′ pairing contribution, local AU contribution, and position contribution. The sum of the context scores for each miRNA was calculated, and the most favorable (lowest) was displayed.

### Microarray

The total RNA was purified using the RNeasy kit (Qiagen) according to the manufacturer's instructions and quantified using the Agilent 2100 Bioanalyzer with RNA 6000 Nano Reagents and Supplies (Agilent Technologies, Inc.). The cRNAs were synthesized and labeled with Cy3 and Cy5, respectively. The labeled cRNA fragments were hybridized with Agilent human 1A 60-mer oligonucleotide microarray, following Agilent 60-mer oligo microarray protocol. Hybridized DNA microarrays were scanned with Agilent 2565BA scanner (Agilent Technologies, Inc.) The raw intensity profiles were analyzed using Feature Extraction Software, and *p*-value LogRatio was used as the criteria to estimate the differences in gene expression.

### Real-time PCR

K562 cells transfected with 100 nM of miR-181a RNA duplex molecule (mimic) or control scrambled miRNA in the presence of Lipofectamine 2000 reagent for 48 h. Total RNA was extracted in Trizol (Invitrogen) and quantified by an ultraviolet spectrophotometer (UVPupland, USA) at a wavelength of 260 nm. cDNA was prepared as described above. The levels of miR-181a and U6 small nuclear RNA (snRNA) were determined by the miRNAs RT-PCR Quantitation Kit (Shang Hai Gene Pharma Company, China). U6 snRNAs were used as the internal control, and the fold change for miR-21 expression level was calculated using the 2^−ΔΔCT^ method.

The RalA mRNAs were determined using SYBR-Green real-time PCR assay. The PCR primers used were 5′-ATCGGAAGAAGGTAGTGC-3′ and 5′-AATCTGCTCC CTGAAGT-3′ (RalA); 5′-CAACGGATTTGGTCGTATT-3′ and 5′-CACAGTCTTCTGG GTGGC-3′ (GAPDH). The levels of RalA mRNA expression was normalized to that of GAPDH.

### Construction of RalA-3′-UTR plasmid and reporter assays

Two oligonucleotides, each contains a RalA miR-181a target site, were synthesized: **5′-CCGctcgagATTGCCTA** ***CATTTATCATGGTCCTGAATGTA*** **GCGTGTAAATT GCCTACAT-3′** (sense strand) and **5′-ATAAGAATgcggccgcTTACACGC** ***T ACATTCAGGACCATGATAAATG*** **TAGGCAATTTACACGCTAC-3′** (antisense strand). The oligonucleotides were annealed and amplified by PCR. This PCR product contained two tandem putative miR-181a binding sites ( **ctcgagATTGCCTA** ***CATTTATCATGGTCCTGAATGTA*** **GCGTGTAAATTGCCTA** ***CATTTATCATGGTCCTGAATGTA*** **GCGTGTAAgcggccgc** , italics stand for miR-181a binding sites,underline is the seed sequence of miR-181a complementary sites) and was cloned into the psiCHECK-2 vector (Promega, Madison, WI) immediately downstream of the Renilla luciferase reporter gene at XhoI and NotI sites (lower case letter) to generate psi-CHECK-*RALA*-UTR [21].

To clone psi-CHECK-*RalA*-mut-UTR containing mutant recognition sequences, two oligonucleotides **5′-CCGctcgagATTGCCTA** ***CATTTATCATGGTCCATGATCAA*** **G CGTGTAAATTGCCTACAT-3′** and **5′-ATAAGAATgcggccgcTTACACGC** ***TTACTAGT GGACCATGATAAATG*** **TAGGCAATTTACACGCTAC -3′** ) were used. The recognition sequence is underlined and the seed sequence of miR-181a complementary sites, **TGAATGT** , was substituted by **TACTAGT** .

Cells were cotransfected with miR-181a together with psi-CHECK-*RALA*-UTR or psi-CHECK-*RALA*-mut-UTR and assayed for luciferase activity 24 h posttransfection using Dual-Luciferase Reporter Assay System (E1910) (Promega). The firefly luciferase activity was normalized with Renilla luciferase activity for each sample.

### Western blot

Cells were lysed in RIPA buffer in the presence of proteinase inhibitor (Biocolor BioScience & Technology company, Shanghai, China). Cell lysates (30 µg) were denatured in Laemmli sample buffer (Bio-RAD) for 5 min at 95.1°C, electrophoresed on 10% SDS-PAGE gel, and transferred to a nitrocellulose membrane. The membrane was blocked with 5% (w/v) fat-free milk in PBS and 0.5% (v/v) Tween-20 for 1 h. The blots were then incubated with anti-RalA antibody (Abcam) at room temperature for 2 h. After washing the blots were then incubated with horseradish peroxidase-conjugated secondary antibody. The signals were visualized with enhanced chemiluminescence (ECL) (BeyoECL Plus, Beyotime company, Haimen, Jiangsu province, China), and analyzed using BI-2000 system.

### Cell growth inhibition

The viability of K562 cells was determined by the 3- (4,5-dimethylthiazol-2-yl) -2,4-diphenyl-tetrazolium bromide (MTT) assay. Briefly, cells were seeded at a density of 1×10^5^ cells/ml in 96-well plates (100 µl/well). The cells were transfected with 181a mimic, or RalA siRNA or control RNAs at a final concentration of 100 nM using Lipofectamine 2000 (Invitrogen) according to the manufacturer's instructions. A total of 50 µl of transfection complexes were added directly to the cells.

At 48 h post-transfection, 20 µl of MTT stock solution (5 mg/ml) was added to each well and the plate was incubated for 4 h at 37°C. The media was then removed and dimethyl sulfoxide (DMSO) (150 µl) was added to dissolve the blue formazan crystals produced by live cells from MTT. Cell viability was assessed by measuring the absorbance at 570 nm on a Bio-Rad microtiter plate reader. Alternatively, the viable cells were also counted using trypan blue exclusion assay in triplicate at 24, 48, and 72 h.

### Flow cytometry

Cells transfected with 181a mimic and/or siRalA (100 nM) were fixed with 70% ethanol were stained with propidium iodide solution (50 µg/ml) containing RNAse A (200 ug/ml). Cell cycle was analyzed using flow cytometry according to the content of DNA. For analyzing apoptosis, cells were stained with FITC-conjugated annexin V and PI. For each sample, data from approximately 10,000 cells were recorded in the list mode on logarithmic scales. Apoptotic and necrotic cells were analyzed by quadrant statistics on propidium iodide (PI)-negative, annexin V-positive cells and PI/Annexin V double positive cells, respectively.

### KEGG analysis

For the RalA pathway analysis, Kyoto Encyclopedia of Genes and Genomes (KEGG) pathway were used to analyze its effector. KEGG is available at the web (http://www.genome.jp/). The result was graphed using cytoscape to show the interactions between RalA and their potential effectors (http://www.cytoscape.org).

### Statistical analysis

All the experiments were carried out in triplicate, and the results were calculated using SPSS10 software asχ±s and statistic analysis was performed using ANOVA. A value of *p*<0.05 was considered to be statistically significant.

## Supporting Information

Table S1 Human miR-181a 691 conserved targets
(XLS)

Table S2 Downregulated genes by microarray
(DOC)
