# Supplementary material for: miR-181a Post-Transcriptionally Downregulates Oncogenic RalA and Contributes to Growth Inhibition and Apoptosis in Chronic Myelogenous Leukemia (CML)
Source: PLoS One. 2012 Mar 19;7(3):e32834. doi: 10.1371/journal.pone.0032834 (PMC3307705; doi:10.1371/journal.pone.0032834)
Supplement: Table S2 — Downregulated genes by microarray (DOC) [file pone.0032834.s002.doc]

**Table.S2. Down-regulated genes by microarray**

| GeneName | Description | PValueLogRatio | Ratio(Cy5/Cy3) |
| --- | --- | --- | --- |
| DRIL2 | Dead ringer (Drosophila)-like 2 | 0.0000 | 0.0084 |
| ABCC6 | ATP-binding cassette subfamily C member 6 | 0.0000 | 0.0362 |
| IFNA4 | Interferon A4, member of the interferon alpha family | 0.0001 | 0.0435 |
| BLVRA | Biliverdin reductase A (biliverdin-IX alpha reductase) | 0.0000 | 0.3537 |
| GJB5 | Gap junction protein beta 5 (connexin 31.1) | 0.0000 | 0.3606 |
| GABRQ | Gamma-aminobutyric acid (GABA) receptor subunit theta | 0.0003 | 0.3631 |
| TADA2L | Transcriptional adaptor 2 like, a transcriptional coactivator | 0.0000 | 0.4361 |
| HEM1 | Hematopoietic protein 1, a member of the HEM family of transmembrane proteins | 0.0002 | 0.4515 |
| SDHA | Succinate dehydrogenase complex subunit A (flavoprotein) | 0.0009 | 0.4676 |
| B3GALT3 | UDP-Gal:betaGlcNAc beta 1,3-galactosyltransferase polypeptide 3 | 0.0007 | 0.4695 |
| NFE2L3 | Nuclear factor erythroid-derived 2 like 3 | 0.0000 | 0.4977 |
| EPN3 | Epsin 3, may be involved in endocytosis and wound healing | 0.0088 | 0.5144 |
| SPAG8 | Sperm associated antigen 8, a sperm plasma membrane antigen | 0.0000 | 0.5184 |
| TNXB | Tenascin XB, a basement membrane protein | 0.0002 | 0.5193 |
| MGC15631 | Protein containing a homeobox domain (homeodomain) | 0.0044 | 0.5297 |
| MFAP2 | Microfibrillar-associated protein 2 | 0.0000 | 0.5346 |
| ATP6V0C | Vacuolar H(+)-ATPase proton pump | 0.0002 | 0.5358 |
| APOA1 | Apolipoprotein A-I | 0.0026 | 0.5386 |
| CD84 | Homo sapiens CD84 antigen (leukocyte antigen) (CD84), mRNA | 0.0000 | 0.5451 |
| CEB1 | Cyclin-E binding protein 1, HECT family member | 0.0000 | 0.5533 |
| PRPSAP1 | Phosphoribosyl pyrophosphate synthetase-associated protein 1 | 0.0000 | 0.5548 |
| PTPN18 | Homo sapiens protein tyrosine phosphatase, non-receptor type 18(PTPN18), mRNA | 0.0000 | 0.5548 |
| NM_015569.1 | Homo sapiens KIAA0820 protein (KIAA0820), mRNA | 0.0000 | 0.5549 |
| AKR1B10 | Aldose reductase-like protein (human small intestine reductase) | 0.0000 | 0.5550 |
| IL13RA2 | Interleukin 13 receptor alpha 2 | 0.0000 | 0.5552 |
| NM_144963.1 | Homo sapiens hypothetical protein FLJ23790 (FLJ23790),mRNA | 0.0000 | 0.5553 |
| IFNA21 | Interferon (alpha 21), a member of the type I interferon family of proteins | 0.0000 | 0.5556 |
| GPR56 | G protein-coupled receptor 56 | 0.0000 | 0.5556 |
| CCL25 | Homo sapiens chemokine ligand 25, transcript variant 2, mRNA | 0.0000 | 0.5556 |
| DERMO1 | Dermo-1 | 0.0000 | 0.5560 |
| NYD-TSPG | Member of the tubulin-tyrosine ligase family | 0.0000 | 0.5561 |
| GPR15 | G protein-coupled receptor 15 | 0.0000 | 0.5562 |
| NEU1 | Neuraminidase 1 (lysosomal neuraminidase, sialidase 1) | 0.0000 | 0.5563 |
| SOD1 | Copper zinc superoxide dismutase | 0.0000 | 0.5567 |
| NM_025214.1 | Homo sapiens CTCL tumor antigen se57-1 (SE57-1), mRNA | 0.0000 | 0.5569 |
| HSD11B2 | Hydroxysteroid 11-beta dehydrogenase 2 | 0.0000 | 0.5573 |
| SLC13A1 | Sodium-sulfate cotransporter 2 | 0.0000 | 0.5577 |
| NUP50 | Nucleoporin, a nuclear pore-associated protein | 0.0000 | 0.5577 |
| TRH | Preprothyrotropin-releasing hormone, precursor of thyrotropin-releasing hormone | 0.0000 | 0.5578 |
| OL-64 | Member of the serpin family of serine protease inhibitors | 0.0000 | 0.5582 |
| KDELC1 | Protein containing a Filamin or Dictyostelium gelation factor repeat domain | 0.0004 | 0.5590 |
| SLC2A5 | Facilitative glucose transporter isoform 5 | 0.0000 | 0.5594 |
| NM_001152.1 | Homo sapiens solute carrier family 25, member 5 (SLC25A5), mRNA | 0.0000 | 0.5596 |
| TAX1BP1 | Tax1 (human T-cell leukemia virus type I) binding protein 1 | 0.0000 | 0.5616 |
| OTX2 | Orthodenticle homolog 2 | 0.0000 | 0.5616 |
| RPP20 | Ribonuclease P subunit p20, an ATPase subunit of a ribonucleoprotein | 0.0000 | 0.5620 |
| NM_152699.1 | Homo sapiens hypothetical protein MGC27076 (MGC27076),mRNA | 0.0000 | 0.5621 |
| KRT9 | Keratin 9 epidermolytic palmoplantar keratoderma, a type I keratin | 0.0001 | 0.5625 |
| EPLIN | Epithelial protein lost in neoplasm | 0.0000 | 0.5633 |
| HLA-A | Homo sapiens major histocompatibility complex, class I, A (HLA-A), mRNA | 0.0000 | 0.5723 |
| CDC2L5 | Cell division cycle 2-like 5 | 0.0000 | 0.5753 |
| SEMA4C | Sema domain, immunoglobulin domain, transmembrane domain and short cytoplasmic domain, (semaphorin) 4C | 0.0010 | 0.5755 |
| TRIP-Br2 | Transcriptional regulator interacting with PHS-bromodomain 2 | 0.0000 | 0.5766 |
| INDO | Indoleamine-pyrrole 2-3 dioxygenase | 0.0000 | 0.5785 |
| AP3S1 | Adaptor-related protein complex 3 sigma 1 subunit | 0.0001 | 0.5786 |
| ARRB2 | Arrestin beta 2 | 0.0000 | 0.5828 |
| KIAA0976 | Protein containing two laminin N-terminal domains and two laminin EGF-like domains | 0.0000 | 0.5840 |
| FRABIN | Protein with strong similarity to rat LOC246174 | 0.0077 | 0.5880 |
| NM_005132.1 | Homo sapiens Rec8p(REC8), mRNA | 0.0006 | 0.5897 |
| DDX18 | DEAD box protein 18 | 0.0017 | 0.5906 |
| PP13 | Placental tissue protein 13 | 0.0001 | 0.5958 |
| NM_138358.1 | Homo sapiens hypothetical protein BC011833 (LOC90580),mRNA | 0.0000 | 0.5969 |
| NM_152407.1 | Homo sapiens hypothetical protein FLJ33918 (FLJ33918),mRNA | 0.0022 | 0.6018 |
| PLA2G2E | Group IIE phospholipase A2 | 0.0000 | 0.6052 |
| LEPREL2 | Protein containing a 2OG-Fe(II) oxygenase superfamily domain | 0.0002 | 0.6053 |
| DSCR1L2 | Down syndrome critical region gene 1-like 2 | 0.0001 | 0.6109 |
| ABCC4 | Homo sapiens ATP-binding cassette, sub-family C, member 4 (ABCC4), mRNA | 0.0008 | 0.6123 |
| PPFIA1 | Homo sapiens protein tyrosine phosphatase,receptor type,f polypeptide,interacting protein,alpha 1,mRNA | 0.0004 | 0.6186 |
| TAF12 | TATA box binding protein (TBP)-associated factor RNA polymerase II J 20kD | 0.0001 | 0.6221 |
| CSTF2T | Protein with strong similarity to cleavage stimulation factor subunit 2 tau | 0.0001 | 0.6234 |
| GNB3 | Guanine nucleotide binding protein beta 3 | 0.0004 | 0.6299 |
| PLCL2 | Protein with high similarity to phospholipase C epsilon (rat LOC84587) | 0.0026 | 0.6331 |
| TK1 | Thymidine kinase 1, a cytosolic form of the enzyme | 0.0002 | 0.6331 |
| NM_022091.1 | Homo sapiens dJ467N11.1 protein (DJ467N11.1), mRNA | 0.0002 | 0.6398 |
| CABP5 | Calcium binding protein 5 | 0.0003 | 0.6446 |
| MRPS5 | Member of the ribosomal S5 family | 0.0016 | 0.6497 |
| CAV2 | Caveolin 2, a member of the caveolin family | 0.0003 | 0.6523 |
| GABRR2 | Gamma-aminobutyric acid receptor rho 2 subunit | 0.0029 | 0.6536 |
| ICT1 | Immature colon carcinoma transcript 1 | 0.0003 | 0.6559 |
| CML66 | Chronic myelogenous leukemia tumor antigen 66 | 0.0003 | 0.6560 |
| KIAA0372 | Protein containing multiple TPR domains | 0.0014 | 0.6569 |
| FBXO32 | F-box only protein 32 (muscle atrophy F-box) | 0.0013 | 0.6580 |
| MTMR1 | Myotubularin related protein 1 | 0.0046 | 0.6582 |
| HDAC9 | Homo sapiens histone deacetylase 9 (HDAC9), transcript variant 2, mRNA | 0.0059 | 0.6590 |
| HADH2 | Hydroxyacyl-Coenzyme A dehydrogenase (type II) | 0.0049 | 0.6618 |
| SIAT4C | Sialyltransferase 4C | 0.0013 | 0.6630 |
| ZNF300 | Protein containing 12 C2H2 type zinc finger domains and a kruppel-associated box domain | 0.0005 | 0.6648 |
| SORT1 | Sortilin 1, non-G protein-coupled membrane receptor for neurotensin | 0.0005 | 0.6660 |
| LMO2 | Lim domain only 2, transcription factor containing a LIM domain | 0.0008 | 0.6660 |
| SYNCOILIN | Intermediate filament protein syncoilin | 0.0005 | 0.6666 |
| HOXA7 | Homeo box A7, a member of the homeodomain-containing family of transcription factors | 0.0012 | 0.6709 |
| RAD51C | RAD51 homolog C (S. cerevisiae), RAD51 family member | 0.0036 | 0.6780 |
| NM_005881.1 | Homo sapiens branched chain alpha-ketoacid dehydrogenase kinase (BCKDK), mRNA | 0.0009 | 0.6780 |
| TDP1 | Tyrosyl DNA phosphodiesterase 1, a member of the phospholipase D superfamily | 0.0032 | 0.6792 |
| VAMP1 | Vesicle associated membrane protein 1 (synaptobrevin 1) | 0.0025 | 0.6803 |
| ZFP28 | zinc finger protein 28 homolog (mouse) | 0.0010 | 0.6811 |
| OGG1 | 8-oxoguanine DNA glycosylase 1 | 0.0046 | 0.6822 |
| MUCDHL | Mucin and cadherin like | 0.0013 | 0.6825 |
| CYGB | Homo sapiens cytoglobin (CYGB), mRNA | 0.0021 | 0.6826 |
| RGR | Retinal G protein-coupled receptor | 0.0042 | 0.6846 |
| RALA | V-ral simian leukemia viral oncogene homolog A (ras related) | 0.0013 | 0.6862 |
| IL18BP | Interleukin 18 binding protein, immunoglobulin superfamily member | 0.0043 | 0.6872 |
| BMPR1B | Bone morphogenetic protein receptor type 1B | 0.0027 | 0.6879 |
| MTNR1B | Melatonin receptor 1B, G protein-coupled melatonin receptor | 0.0026 | 0.6882 |
| PLCB2 | Phospholipase C beta 2 | 0.0073 | 0.6893 |
| NM_022097.1 | Homo sapiens hepatocellular carcinoma antigen gene 520 (LOC63928), mRNA | 0.0030 | 0.6895 |
| MYH3 | Embryonic skeleltal muscle myosin heavy polypeptide 3, a member of the myosin family | 0.0089 | 0.6901 |
| ERBB4 | Avian erythroblastosis oncogene B 4 | 0.0020 | 0.6904 |
| ALOX12B | 12R-lipoxygenase | 0.0069 | 0.6910 |
| CNOT4 | Homo sapiens CCR4-NOT transcription complex, subunit 4, mRNA | 0.0018 | 0.6935 |
| FLJ20313 | Protein with low similarity to myotubularin-related protein 8 | 0.0025 | 0.6957 |
| KREMEN2 | Protein with strong similarity to kringle containing transmembrane protein 2 | 0.0022 | 0.6998 |
| S100A13 | Homo sapiens S100 calcium binding protein A13 (S100A13), mRNA | 0.0023 | 0.7010 |
| FLNC | Filamin C gamma | 0.0054 | 0.7019 |
| FLJ10581 | Member of the RNA methyltransferase family | 0.0077 | 0.7019 |
| BF | B-factor, a serine protease | 0.0071 | 0.7025 |
| SCNN1A | Sodium channel (nonvoltage-gated) channel 1 alpha | 0.0084 | 0.7049 |
| HBA1 | Hemoglobin alpha 1, a component of tetrameric hemoglobin A(1) | 0.0035 | 0.7085 |
| RELB | Reticuloendotheliosis viral (v-rel) oncogene related B | 0.0093 | 0.7155 |
| RBM3 | RNA binding motif protein 3 | 0.0040 | 0.7158 |
| MGC33182 | Protein with strong similarity to casein kinase 1 alpha 1 | 0.0093 | 0.7160 |
| RAD50 | RAD50 homolog, a subunit of the double-strand break repair complex | 0.0075 | 0.7171 |
| LEPRE1 | Leucine proline-enriched proteoglycan (leprecan) 1 (growth suppressor 1) | 0.0058 | 0.7202 |
| STAU | Staufen, binds dsRNA, RNA-DNA hybrids, and tubulin | 0.0091 | 0.7203 |
| NM_015141.1 | Homo sapiens KIAA0089 protein (KIAA0089), mRNA | 0.0048 | 0.7209 |
| FLJ13590 | Protein with high similarity to zinc finger and BRCA1-interacting protein | 0.0050 | 0.7223 |
| SYTL4 | Protein with strong similarity to synaptotagmin-like 4 (granuphilin, mouse Sytl4) | 0.0078 | 0.7245 |
| SPARC | Secreted protein acidic cysteine-rich (osteonectin), a glycoprotein | 0.0086 | 0.7248 |
| LQFBS-1 | Bat2 HLA-B-associated transcript (KIAA0515) | 0.0059 | 0.7270 |
| ASF1A | Member of the anti-silencing protein ASF1-like family | 0.0084 | 0.7315 |
| ACTL7B | Actin-like 7B, a member of the actin-related protein (ARP) family | 0.0080 | 0.7350 |
| RAP1GDS1 | Rap1 GTPase-GDP dissociation stimulator 1 | 0.0093 | 0.7372 |
| RALBP1 | Retired, replaced by A_23_P376599, was RalA binding protein 1 | 0.0002 | 0.6246 |
